# Effects of light-load vs. heavy-load jump squats as priming activities in Olympic female rugby sevens players

**DOI:** 10.5114/biolsport.2025.142645

**Published:** 2024-09-06

**Authors:** Irineu Loturco, Piotr Zmijewski, Valter P. Mercer, Maurício S. Ramos, Marina T. Betelli, Ismael Arenhart, Túlio B. M. A. Moura, Lucas A. Pereira

**Affiliations:** 1 NAR – Nucleus of High Performance in Sport, São Paulo, Brazil; 2 Department of Human Movement Sciences, Federal University of São Paulo, São Paulo, Brazil; 3 University of South Wales, Pontypridd, Wales, United Kingdom; 4 Jozef Pilsudski University of Physical Education in Warsaw, Warsaw, Poland; 5 Research and Development Center Legia Lab, Legia Warszawa, Poland; 6 CBRu – Brazilian Rugby Confederation, São Paulo, Brazil

**Keywords:** Team-sports, Athletic performance, Resistance training, Potentiation, Ballistic exercises

## Abstract

Priming activities have been widely used by coaches as a strategy to enhance physical performance within short periods (6–24 hours) before sport-specific training sessions and competitions. In this crossover study, we examined and compared the effects of two different priming schemes on the speed-power performance of female rugby sevens players. One hour after completing a standardized warm-up and a series of measurements including loaded and unloaded jumps and speed-related tests, twenty Olympic female rugby sevens players performed, one week apart, 6 sets of 6 reps of jump-squats (JS) at either 40% (light-load; LL) or 80% 1RM (heavy-load; HL). Countermovement jump height increased significantly 6-h after both loading conditions (ES=0.50 and 0.34, for LL and HL, respectively; *P* < .001), with no changes observed at the 24-h time-point. JS peak velocity improved significantly after 24-h compared to the pre-testing, but solely for the lighter loading intensity (i.e., JS at 40%1RM; ES=0.63; P=0.006). 40-m sprinting speed increased significantly at the 6-h timepoint for both LL (ES=0.20; P=0.001) and HL (ES=0.18; P=0.004), without showing significant changes in the following 24-h. COD speed improved significantly after both priming schemes at the 6- and 24-h time points, regardless of the loading condition (P ≤ 0.027 for the main effect of time). No time × loading condition interaction was detected for any variable assessed, with P-values ranging from 0.111 to 0.953. Importantly, the rate of perceived exertion was significantly higher after the priming protocol at the HL condition (P=0.02), which may lead to increased levels of fatigue and decreased performance in subsequent activities. Elite coaches from rugby sevens (and other team sports) should strongly consider these findings when programming priming training sessions in the periods preceding more intensive training sessions and official matches due to the potential disadvantages associated with the use of heavier loads (i.e., ≥ 80% 1RM).

## INTRODUCTION

In any sport, coaches and practitioners are always seeking practical and effective approaches to significantly improve physical and technical performance [1–4]. In this regard, both chronic (e.g., strengthpower training schemes) and acute (e.g., post-activation potentiation enhancement; [PAPE]) methods have been extensively investigated in sport sciences [5–7]. Besides the more traditional resistance training strategies (e.g., concentric and eccentric strength training), alternative training arrangements such as complex training, contrast training, eccentric training, and training within the optimum power zone, have been widely analyzed and used in real-world sport contexts [8–11]. Along these long-term programs, acute (e.g., PAPE) or even other types of short-term interventions have been frequently tested by coaches and their technical staff during training and competitions, with some of them showing promising results.

In this context, “priming exercises” (i.e., strategies prescribed by practitioners to top-level athletes prior to competition to assist preparation and intentionally enhance athletic performance [12]) have become a highly researched topic in our field [12–14]. A survey study by Harrison et al. [12] revealed that elite coaches typically include 2–3 lower-body and 2–3 upper-body strength-power exercises involving various forms of jumping, squatting, pressing, and pulling movements, preferably executed with maximal or near-maximal velocity intent. Despite the great variability in exercise selection, loading intensities (i.e., percentages of one-repetition maximum; [% 1RM]) and training methods adopted by ≈70 coaches from different sports (e.g., rugby, soccer, track &field, and field hockey), priming activities are usually prescribed within the 0–8-h period before competitions (a preference reported by ≈60% of these coaches). Longer periods of time (from 9 to 32 hours) represent approximately 36% of coaching preferences [12], with only a minority of coaches (≈15%) utilizing recovery periods longer than 24-h. These practices, many of them developed and supported by personal coaching experiences, have recently been examined by researchers and sport scientists interested in improving subsequent sport-specific performance.

In general, priming interventions have typically been prescribed and implemented utilizing heavy loads (i.e., > 80% 1RM) and more traditional resistance exercises (e.g., parallel squat and benchpress) [15, 16], or ballistic lifts executed under moderate loading conditions (i.e., jump squats [JS] at 40% 1RM) [17]. Priming activities using other types of resistance training protocols (e.g., variable resistance training with the use of elastic bands) have also been tested in state-level rugby players, showing divergent results in terms of upper- and lower-limb performances [18]. Notwithstanding the short recovery time employed in that study (i.e., 1-h 45-min), the upperbody peak power and peak force (both assessed in the bench-throw exercise) increased significantly after the priming activity, which comprised 4 sets of 3 reps of either bench presses or back squats [18]. Nevertheless, no changes occurred in any of the countermovement jump (CMJ) measures, suggesting that, at least for the variables and protocols evaluated in this study, the priming effects were not evident for lower-body power or force expression. Likewise, Harrison et al. [19] did not observe any significant increases in upper-body strength-power qualities (i.e., bar-peak velocities assessed in maximal bench throw and bench pull tests at 25%, 50%, and 75% 1RM) in resistance-trained subjects 3- and 27-h after completing a priming stimulus involving 4 sets of 3 reps of bench press and bench pull exercises executed at 65% 1RM. These initial findings indicate that, regardless of the time course or exercise stimuli, positive changes in physical performance are difficult to achieve through the use of typical or even atypical (i.e., variable resistance training) priming strategies [18].

Conversely, Saez de Villarreal et al. [16] revealed that the use of a modified warm-up protocol comprising heavy-load exercises (i.e., 2 sets of 4 reps, 2 sets of 2 reps, and 2 sets of 1 rep at 80%, 90%, and 95% 1RM, respectively, in the parallel squat exercise) performed along with a specific volleyball warm-up protocol involving multiple tasks and jumping drills may result in larger acute gains in leg power-related qualities (e.g., loaded, unloaded, and drop jumping heights) of competitive male Spanish first division volleyball players. Interestingly, these effects could be maintained even after long recovery periods (e.g., 6-h) when prior high-intensity dynamic actions (i.e., 3 sets of 5 loaded jumps using the load that maximizes power output [optimum load] [11]) were applied in the previous warm-up session. Similarly, Cook et al. [15] used a maximum sprint-strengthbased protocol (i.e., 5 maximum sprints of 40 m or 3 RMs bench presses and back squats) in the morning (i.e., 9:00 AM) to assess and compare the physical performance of semi-professional rugby union players in the afternoon (i.e., 3:00 PM). Overall, players displayed improved jump power, sprint speed, and greater increases in 3RM load in both bench press and back squat exercises after completing the resistance training stimulus. They also achieved faster sprinting speeds after executing the maximum sprint protocol [15], but only when compared with the control group (i.e., “rested players”). Based on these previous findings, it can be inferred that: 1) athletes with superior training backgrounds or higher levels of performance (e.g., professional 1^st^ division volleyball players or semi-professional rugby players) may benefit more from priming strategies, irrespective of the period between the priming exercises (e.g., from 5-min to 6-h) and the target competition or specific training session [16, 18]; 2) priming activities involving multiple sets of a given exercise (e.g., squats) executed at varying training intensities (i.e., 80–95% 1RM; 3RM load) or including successive sets of loaded jumps (i.e., using additional loads relative to the subjects’ body mass; [BM]) may lead to positive outcomes in a range of independent measures of athletic performance, such as jumping power and height, sprint speed, and maximum strength [15, 16].

Considering the scarcity of studies on priming exercises with toplevel athletes, especially at the Olympic level, it is essential to determine the potential effects of this strategy on their speed and power qualities. Additionally, given that some improvements in physical performance have been observed after longer time intervals (e.g., ≥ 6-h) it is important to re-test these athletes at even longer recovery periods (e.g., 24-h). This would be particularly valuable for coaches when planning training sessions in the days preceding official competitions. This study aimed to evaluate the effectiveness of two different priming activities involving an exercise commonly used in professional training settings (i.e., JS), performed at two distinct loading conditions (i.e., 40% vs. 80% 1RM) on a series of speed- and power-related capabilities of Olympic female rugby sevens players, assessed 6 and 24 h after completing the priming sessions.

## MATERIALS AND METHODS

### Participants

Twenty Olympic female rugby sevens players (age: 22.9 ± 4.1 years; BM: 67.1 ± 6.9 kg; height: 1.69 ± 0.05 m) from the Brazilian National Team participated in this study. Players were tested in the final phase of preparation for the “World Rugby Sevens Series”. The typical training program of the players at this stage is detailed in Table 1. The study was approved by the local Ethics Committee, and all subjects were informed of the inherent risks and benefits associated with study participation before signing informed consent forms.

**TABLE 1:** Typical weekly training program for the Olympic female rugby players during this respective preparation phase.

	Monday	Tuesday	Wednesday	Thursday	Friday
***Morning***	IPP 30’ Resistance training 60’ TEC/TAC 60’	IPP 30’ Resistancetraining 60’ TEC/TAC 60’	IPP 30’ Resistance training 60’ TEC/TAC 60’	IPP 30’ Resistance training 60’ TEC/TAC 60’	IPP 30’ Resistance training 40’ TEC/TAC 45’
***Afternoon***	TEC (individual skills) 30-40’	Conditioning training 30-40’	Rest	Conditioning training 30’ TEC (individual skills) 30’	Conditioning training 30’

IPP = injury prevention practices; TEC = technical training; TAC = tactical training. Numbers after training sessions represent the total volume in minutes. TEC/TAC training involved various formats of game-based training and specific technical-tactical actions (e.g., shooting, passing, tackling, mauling, and contact events, etc.). Injury prevention practices involved joint mobility exercises, dynamic stretching, core exercises, and elastic band movements. Resistance training involved traditional exercises (e.g., half-squat, Olympic weightlifting derivatives, bench press, prone row, and push press) ballistic exercises (e.g., loaded jumps and medicine ball throw), with loads ranging from 70 to 90% of 1RM for traditional exercises and from 30 to 60% 1RM for ballistic exercises. Plyometrics (e.g., drop and hurdle jumps, and bounding) were also executed during complex (resistance training sessions) under unloaded conditions. Conditioning training comprised different protocols of rugby-specific high-intensity interval training, skill-based conditioning training, etc.

### Study Design

**FIG. 1:**
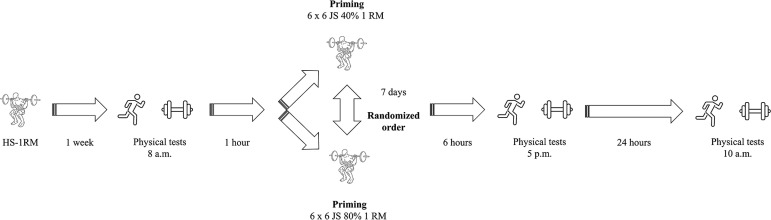
Schematic presentation of the study design. HS: half-squat; 1RM: one-repetition maximum; JS: jump squat.

**FIG. 2:**
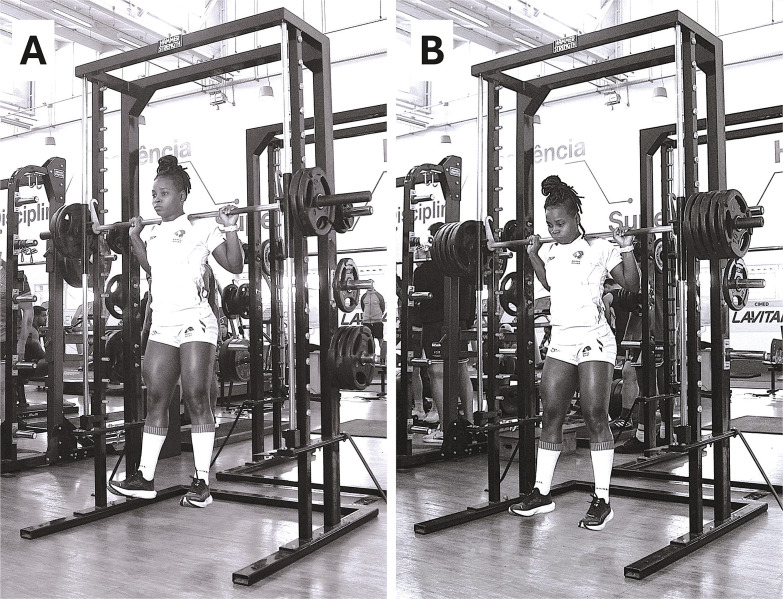
A National female rugby sevens player performing the jump squat exercise during the priming activities at light (JS at 40% 1RM) and heavy (JS at 80% 1RM) loading conditions (Panels A and B, respectively).

This randomized crossover study tested two distinct priming strategies on the speed and power performance of Olympic rugby sevens players. The study design is described in Figure 1. Athletes were assessed over three consecutive weeks and performed all testing sessions and priming activities at their regular training times, adhering to their professional training routines and by the National coaching staff. In the first week, the 1RM was determined in the half-squat (HS) exercise. In the subsequent weeks, the experimental procedures were progressively implemented. Players arrived at the high-performance training centre and, after completing a standardized warm-up involving general (i.e., 10-min running at a moderate pace, rated as 3 on the 0–10 rating of perceived exertion (RPE) scale and dynamic stretching) as well as movement-specific exercises (i.e., submaximal attempts of sprint-jump exercises), the physical tests were executed in the following order: CMJ, sprinting speed at 10- and 40-m, 505 change of direction (COD) speed test, and measurement of the peak velocity (PV) in the JS exercise. After a one-hour rest, players executed the priming activities, which consisted of performing JS at either 40% or 80% of HS 1RM (Figure 2), in a randomized order.

The physical tests were repeated after 6- and 24-h intervals. During this period, athletes were not engaged in any training sessions. One week later, the procedures were repeated in a crossover fashion. Throughout this week, the Olympic female rugby players were involved in low-intensity motor tasks and exercises comprising technical training (i.e., passing drills, ball reception, and shooting accuracy), physical therapy and other health care services, recovery procedures, nutritional assessments and body composition tests, and personal meetings with the psychologists. After the priming protocols under both light (JS at 40% 1RM) and heavy (JS at 80% 1RM) loading intensities, RPE was assessed using the Borg category ratio (e.g., 0–10) scale as adapted by Foster et al. [20].

### Procedures

#### One-Repetition Maximum in the Half-Squat Exercise

Maximum strength was assessed using the HS 1RM test, as previously described and adapted [21]. Before the test, players performed a standardized warm-up, which consisted of 5 repetitions ranging from 40% to 60% of the estimated 1RM. Three minutes after the warm-up, players were allowed up to 5 trials at approximately 70%, 80%, 90%, and > 95% of the estimated 1RM to determine the 1RM value [21, 22] accurately. A 3-minute rest interval was allowed between the sequential and incremental attempts [21, 22]. Athletes were required to move the barbell as fast as possible across the concentric phase of the lift in all trials.

### Countermovement Jump Test

Vertical jump height was assessed using the CMJ. Athletes were required to execute a downward movement followed by complete extension of the legs and were free to determine the countermovement amplitude to avoid changes in jumping coordination [21]. All jumps were performed with the hands on the hips, and the athletes were instructed to jump as high as possible. The jumps were conducted on a contact platform (Elite Jump^®^, S2 Sports, São Paulo, Brazil), and jump height was automatically calculated using the flight-time method. A total of five attempts were allowed, with 15-s intervals between the consecutive CMJ trials. The best attempt was used for subsequent analyses.

### Sprinting Speed

Sprint testing was conducted on an indoor running track. Three pairs of photocells (Elite Speed System^®^; S2 Sports, São Paulo, Brazil) were positioned at the starting line and at distances of 10- and 40-m. Players sprinted twice, starting from a standing position 0.5-m behind the starting line. Sprinting speed was calculated as the distance travelled over a measured time interval. A 5-minute rest interval was allowed between sprint trials, and the fastest time was considered for analysis.

### 505 Change of Direction Test

Players started from a standing position with the front foot placed 0.5 m behind the starting line, and a pair of photocells was positioned at a distance of 10 m (Elite Speed System^®^; S2 Sports, São Paulo, Brazil). Players were required to sprint to the COD area (positioned at 15 m), place their foot on the line, perform a 180º turn, and sprint back through the finishing line (i.e., 10-m timing gate). The time from the 10-m timing gate to the COD area and back to the 10-m gate was recorded for the 505 COD time. The 505 COD speed was calculated as the distance travelled over a measured time interval. Athletes sprinted twice for each side, with a 5-minute rest interval between attempts, and the fastest time was considered for analysis.

### Peak Velocity in the Jump Squat Exercise

The PV was assessed in the JS exercise using loads corresponding to 40% and 80% of the HS 1RM. The test was conducted on a Smith-Machine device (Hammer Strength Equipment, Rosemont, IL, USA). Players were required to execute three repetitions for each respective load at maximal intended velocity, with 15-s intervals between repetitions. To record and determine the PV, a linear velocity transducer (T-Force, Dynamic Measurement System; Ergotech Consulting S.L., Murcia, Spain) sampling at 1000 Hz was attached to the barbell. The highest PV at each load was used for analysis.

### Statistical Analysis

Data are presented as mean ± standard deviation. Data normality was checked using the Shapiro-Wilk test. To test for differences between the RPE obtained after the priming activities performed with different loads, an independent t-test was conducted. A two-way ANOVA with repeated measures (time × loading condition interaction), followed by the Bonferroni’s post-hoc test, was used to test for differences over the distinct time points between light and heavy loading conditions. The level of significance was set at *P* < 0.05. To determine the magnitude of the differences among the measurements executed across the distinct time points, effect sizes (ES) were calculated using Cohen’s *d*[23] to indicate the magnitude of changes and interpreted using the thresholds proposed by Rhea [24] for highly-trained subjects, as follows: < 0.25, 0.25–0.50, 0.50–1.00, and > 1.00 for trivial, small, moderate, and large, respectively. Absolute and relative reliability were assessed using the coefficient of variation (CV) and the two-way random intraclass correlation coefficient (ICC), respectively.

## RESULTS

**FIG. 3:**
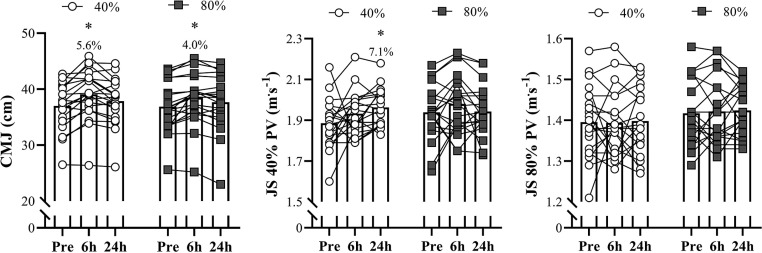
Comparisons of the countermovement jump (CMJ) and barbell peak velocity (PV) in the jump squat (JS) exercise at 40% and 80% 1RM between the different priming activities and across different testing sessions. *P < 0.05 in relation to pre-values.

All measurements utilized in this study exhibited high levels of absolute and relative reliability (i.e., ICC > 0.90 and CV < 10%). No significant differences between light and heavy loading conditions were observed for any variables assessed in the pre-testing session (*P* > 0.05). Figure 3 shows the comparisons of the CMJ, and barbell PV at 40% and 80% 1RM between the different priming activities and across the different testing sessions. Higher CMJ height was observed 6-h after both priming sessions (ES – 0.50 and 0.34 for 40% and 80% 1RM, respectively; *P* < .001 for both loads). No significant changes were observed in CMJ performance 24-h postpriming for both loading conditions (ES – 0.20 and 0.14 for 40% and 80% 1RM, respectively; *P* > 0.05). No significant changes in PV at 40% and 80% 1RM were detected after 6-h and 24-h postpriming sessions for any loading condition (ES ranging from 0.02 and 0.39; *P* > 0.05), except the JS 40% 1RM session, which showed a higher PV at 40% 1RM after 24-h compared to the pre-test (ES – 0.63; *P* – 0.006). No time × loading condition interaction effect was detected for CMJ (*P* – 0.607), and PV at 40% 1RM (*P* – 0.111) and 80% 1RM (*P* – 0.953).

**FIG. 4:**
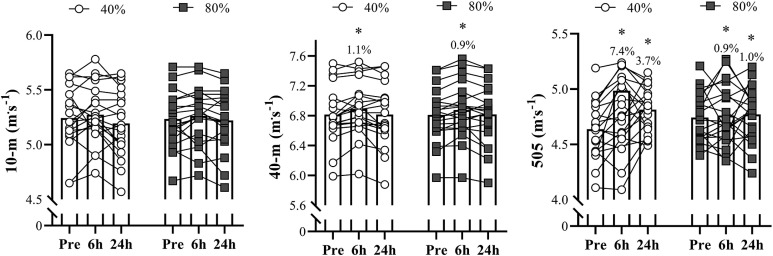
Comparisons of the linear sprint and 505 change of direction speed tests between the different loading conditions across the physical testing sessions. *P < 0.05 in relation to pre-values.

Figure 4 depicts the comparisons of the linear and 505 COD speed tests between the different loading conditions across the physical testing sessions. No significant changes were observed in the 10-m sprint speed after the completion of the priming exercises for any of the loading conditions (ES ranging from 0.03 to 0.18; *P* > 0.05). The 40-m sprint speed increased at the 6-h time-point for both 40% and 80% 1RM conditions (ES – 0.21 and 0.20; *P* – 0.001 and 0.004, respectively), with no significant differences detected at 24-h for both intensities (ES – 0.01 for both loads; *P* > 0.05). The COD speed improved significantly at the 6-h and 24-h time points for both priming schemes, irrespective of light or heavy loading conditions (ES – 1.34 and 0.68; *P* – 0.027 and < .001 after the 6- and 24-h JS 40% 1RM session, respectively; ES – 0.20 and 0.21; *P* – 0.027 and < .001 after 6- and 24-h JS 80% 1RM session, respectively). No time × loading condition interaction effect was detected for 10-m (*P* – 0.527), 40-m (*P* – 0.923), and COD (*P* – 0.198) speed tests.

**FIG. 5:**
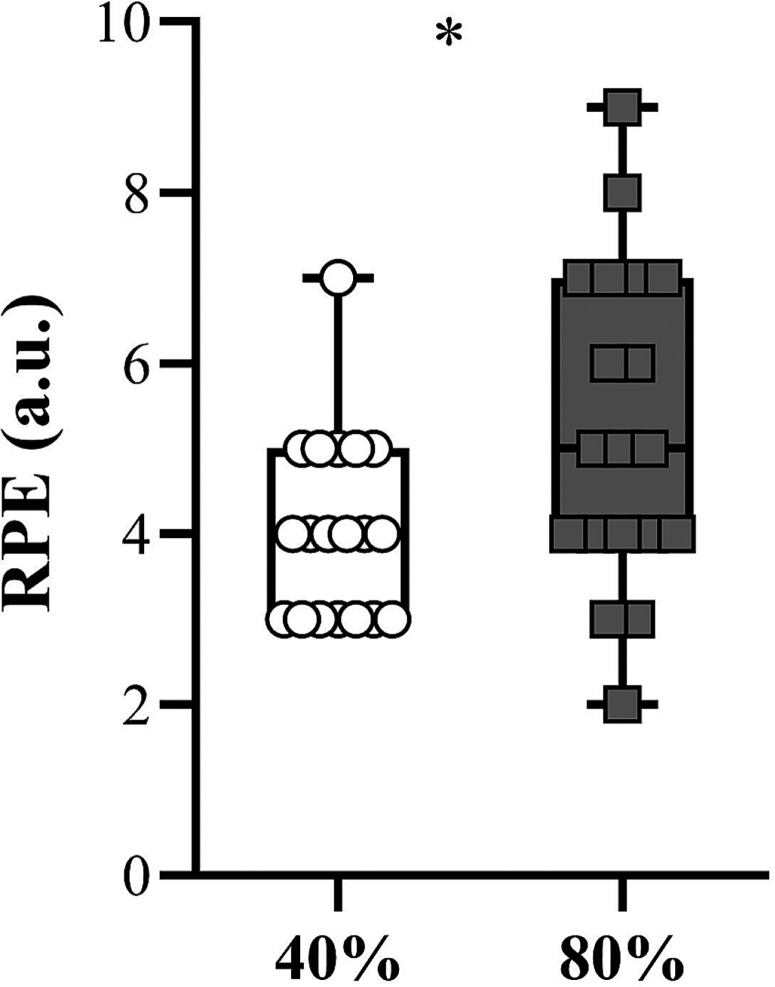
Comparison of the rating of perceived exertion (RPE) obtained after the priming activities performed under the different loading conditions. *P < 0.05.

Figure 5 shows the comparison of the RPE obtained after the priming exercise sessions performed with the different loading conditions. Players reported a higher RPE after the 80% 1RM condition compared to the session executed at 40% 1RM (*P* – 0.02).

## DISCUSSION

In this crossover study, we examined the effects of two different priming strategies (i.e., JS executed at light vs. heavy loads) on the speed- and power-related capabilities of Olympic female rugby sevens players. Overall, the female players displayed superior CMJ performance 6-h after both priming sessions, with no increase in CMJ height observed after 24-h. Regarding PV, no significant changes were detected in any group at the 6-h testing session; however, the JS 40% 1RM condition demonstrated a higher PV at 40% 1RM 24-h after the pre-tests. Sprinting speed increased only at the 6-h testing session for both the 40% and 80% 1RM loading conditions, but this increase was observed solely for the 40-m distance (and not for the 10-m distance), without significant differences between light and heavy loads. Notably, COD speed – a very important physical quality for rugby sevens [25] – improved significantly for both loading conditions and at both time points (i.e., after 6-h and 24-h). Lastly, and not surprisingly, the female sevens players reported a higher RPE after the priming activity performed with heavier loads (i.e., JS at 80% 1RM) compared with the session performed with lighter loads (i.e., JS at 40% 1RM). Together, this information has important implications for rugby sevens coaches who commonly prepare their athletes to play a series of official matches (e.g., ≈6–8 matches) within very short periods (e.g., 2 days) during international tournaments (e.g., “Women’s Sevens World Series”).

To some extent, our findings are similar to those reported by Cook et al. [15], who reported increases of ≈3% in CMJ peak power 6-h after completing a priming activity consisting of back squats and bench presses using a “3 RM load” with male semi-professional rugby players. Another study conducted with sport science students compared the effects of training “to failure” vs. half the maximum number of repetitions per set (i.e., 3 sets of 8 reps out of 8 possible reps vs. 3 sets of 4 reps out of 8 possible reps, respectively) and revealed a decrement of ≈8% in CMJ height 6-h after performing the maximum number of repetitions that can be completed within the set [26]. In a research including 19 National youth Olympic weightlifters (with 6 of these athletes previously representing the US team in international competitions) Fry et al. [27] observed that 5 sets of 3 repetitions of clean and snatch lifts executed at submaximal loads (i.e., 85% 1RM) enhanced vertical jump height by ≈5% in the “responders” group (i.e., athletes with higher levels of anxiety before competition [28]) approximately 6-h after this activity, immediately before a simulated contest. Hence, at least for more experienced participants (i.e., semi-professional or professional athletes) some benefits in jumping ability (and perhaps in other power-related abilities) may be achieved through the use of a few sets of ballistic exercises.

As mentioned above, neither light nor heavy loading conditions influenced the CMJ performance of our players in the 24-h following the priming stimuli. Somewhat surprisingly, there is a gap in studies on priming responses involving resistance training schemes (e.g., using loaded jumps or back squats) that have assessed elite athletes, especially women, at the 24-h time-point. However, these investigations predominantly comprised male subjects, which significantly limits the comparison with our results. In a cross-over study carried out over a 7-day period, Howatson et al. [29] did not find any change in the CMJ height of track and field athletes (6 men and 4 women) recruited from the UK Olympic Performance Centre, after comparing the effects of short “maximum strength” vs. “power” training sessions (i.e., 3 types of squat exercises, each consisted of 4 sets of 5 reps; loads determined by the RPE scale, a common method used by elite UK track and field athletes [30]) 24-h after completing the priming exercises. Other studies with similar designs conducted with subjects with different training backgrounds (i.e., lesser-trained participants, physically active sport science students, resistance trained athletes) [26, 31, 32] also showed no changes or even decreases in CMJ height at the 24-h time point, which, along with our findings (Figure 3) (lack of improvements in CMJ performance), questions the use of priming activities 24-h before participating in more intensive training sessions and, particularly, competitions. It is worth noting that there is a trend towards worse performances when these activities are performed to failure or with heavier loading conditions (e.g., ≥ 85% 1RM) [26, 32].

The PV attained against loads relative or absolute loads is an important indicator of physical performance [33, 34]. Generally speaking, considering the close relationship between relative load and movement velocity [35], we can infer that subjects who can move 40% and 80% of 1RM faster after any type of acute or chronic intervention are also able to apply greater amounts of force against other maximal or submaximal loads (including at the 1RM load) [35, 36]. Therefore, the significant increment (+ 7.1%) (Figure 3) in PV at 40% 1RM observed 24-h after the “light” (JS at 40% 1RM) priming activity may be extremely relevant in athletic contexts where force and power application at higher velocities is necessary as, for example, in rugby sevens [37, 38]. Conversely, the absence of significant enhancements in barbell PV at the 6-hour time point may indicate that this shorter recovery period is insufficient to induce positive changes in power- or force-related outputs measured during this assessment interval. Ekstrand et al. [39] reported similar results in CMJ peak power 4–6-h after prescribing a “two-part typical workout used in training for power sports” for a sample composed of 14 throwers (8 men and 6 women), including several US National caliber throwers. Briefly, after performing sets of power clean (executed in a fast and explosive manner) and back squats under a wide range of submaximal loads, throwers completed a set of back squats to failure, with a load corresponding to 85% 1RM. Partially, and according to our results, this might suggest that, irrespective of the exercise being performed, a combination of heavy loads (i.e., ≥ 80% 1RM) and sets to failure may be ineffective in improving power-related measures within a 6- to 24-h period, even when dealing with highly-trained subjects [32, 39, 40]. In contrast, and also aligned with our outcomes, Nishioka and Okada [41] found that resistance priming activities using a light-load ballistic exercise (i.e., 5 sets × 4 reps of JS at 40% 1RM) enhanced force output at high velocity during vertical jumping trials. This finding is quite compatible with the substantial increments of ≈7% achieved by our Olympic female rugby sevens players who executed JS under similar loading conditions – an increase that was not observed with heavier loads (i.e., 80% 1RM).

Perhaps one of the most important results of this research is that both 40-m sprint speed and COD speed improved significantly following both light and heavy priming protocols: at 6-h for linear sprinting and at 6- and 24-h time-points for COD speed. Curiously, the majority of studies investigating the influence of priming exercises on sprinting performance utilized distinct forms of priming activities (e.g., resisted sprints, unloaded sprints) rather than traditional resistance training schemes [15, 42]. For example, Bachero-Mena et al. [42] reported significant decreases in 20-m sprint speed after prescribing 8 sets of 20-m resisted sprints with 80% BM in a study with male sport science students, who used sled loads from 20% to 80% BM. On the other hand, at post-24 h, sprinting speed remained unchanged for all loading conditions (resisted sprints at 20%, 40%, 60%, and 80% BM). According to the authors, these results indicate that higher sled loads may cause greater impairments in physical performance immediately after testing, principally when utilizing very-heavy sled loads (i.e., 80% BM) during resisted sprint training sessions. Cook et al. [15] demonstrated a significant improvement in 40-m sprint performance 6-h following the completion of 5 sets of 40-m sprints in semi-professional rugby players. Despite the differences with our protocol (i.e., JS-based vs. sprint-based exercises), Cook’s study [15] also reveals that the sprint speed of elite athletes may be enhanced when a few sets (i.e., 3–4) of ballistic lifts or maximal unloaded sprints are executed within short-intervals (i.e., ≈6-h) prior to sport-specific training sessions or matches. It is essential to highlight that, in our study, the speed increase achieved by the lighter condition was slightly higher than that achieved by the heavier loading condition (i.e., 1.1% vs. 0.9%, respectively) (Figure 4).

Even though these latest differences in sprint speed may seem small, they gain greater importance when observing and comparing the increases in COD speed that the Olympic female rugby sevens players obtained at 6- and 24-h time points (7.4% and 3.7% vs. 0.9% and 1.0%, respectively, for JS at 40% and at 80% 1RM, 6- and 24-h after completing the priming activity). Among a series of factors, these results confirm the relevance of prioritizing light-loading conditions (and faster movement velocities) when the intention is to maximize force and power production at higher velocities with the use of priming strategies (i.e., movement velocity specificity) [41, 43]. As mentioned earlier, this aspect could be even more pronounced in highly-trained (i.e., stronger) individuals [41, 43]. To optimize these positive responses, the use of ballistic lifts (i.e., exercises that exclude any deceleration phase throughout the entire range of motion [44]) is strongly recommended, specifically when the intention is to enhance athletic performance within periods lasting up to 24-h [41, 43]. Indeed, the mechanical advantages of these movements over the more traditional resistance exercises have been extensively documented in the literature [21, 44–47], as well as the importance of prescribing them under light-to-moderate loading conditions (e.g., 20–60% 1RM) [21, 48].

Last but not least, the heavier loading condition (i.e., JS at 80% 1RM) resulted in a much higher RPE than the lighter condition (i.e., JS at 40% 1RM) (Figure 5), an aspect that was also reported by the coaching staff of the Brazilian National Team (*personal communication*). Along with the actual barbell velocity (which is much faster and thus, less exhausting, at 40% 1RM compared to 80% 1RM), it is rational to consider that sets of 80% 1RM with a proportional number of repetitions will lead to higher percentages of velocity loss. This training configuration will undoubtedly induce greater levels of metabolic and neuromuscular fatigue, thereby hampering or at least reducing subsequent acute or medium-term (e.g., 24–48-h) performance [26, 49].

This research has several limitations, most of them related to the fact that the study has been completed during the last stage of the Olympic cycle (which, on the other hand, may be seen as a great advantage). An essential point is that the authors’ team decided not to discuss mechanisms or potential neuromuscular issues related to our main outcomes and priming responses. This decision was based on three consistent and applied views: 1) there is plenty of evidence about these (and other directly related) aspects in the literature [13, 14, 50], which may support our findings and elucidate potential questions regarding our methods and results; 2) this is the first study on priming activities conducted with Olympic female teamsport athletes already qualified for the Olympic Games, highlighting the importance, interest, and scarcity of such data; 3) the option to produce and prepare a practical research that can guide and provide useful insights for coaches and practitioners specifically involved in Olympic Games and International Tournaments. Further studies are required to explore the neuromechanical origins and underlying mechanisms of the priming responses described here. In future research, it would be valuable to compare the effects of other priming interventions, such as low-volume sessions involving heavy sled pushes (or using non-failure or moderate velocity loss resistance training protocols with moderate-to-heavy loads), on delayed potentiation in sprint performance and countermovement jumps [51].

## CONCLUSIONS

The clear indication that heavier loads do not present any advantage over lighter loads and even cause higher RPE and lower increases in speed- and power-related qualities when used as priming activities between 6-h and 24-h before sport-specific training sessions and matches should be strongly considered by rugby sevens coaches (as well as by coaches from other team-sports). When correctly applied (i.e., using light-loads and low sets of ballistics lifts such as the JS), these priming schemes may improve vertical jumping and power-related qualities, as well as eliciting significant increments in linear sprinting and COD speed. Although the time-course of these physical improvements might vary between 6- and 24-h, there is no doubt that these strategies may help players and their respective teams to achieve higher levels of physical (and perhaps technical) performance during more intensive training sessions and official competitions.
